# Micro-mechanical insights into the dynamics of crack propagation in snow fracture experiments

**DOI:** 10.1038/s41598-021-90910-3

**Published:** 2021-06-03

**Authors:** Grégoire Bobillier, Bastian Bergfeld, Jürg Dual, Johan Gaume, Alec van Herwijnen, Jürg Schweizer

**Affiliations:** 1 WSL Institute for Snow and Avalanche Research SLF, Davos, Switzerland; 2 Institute for Mechanical Systems, ETH Zurich, Zurich, Switzerland; 3 SLAB Snow and Avalanche Simulation Laboratory, EPFL Swiss Federal Institute of Technology, Lausanne, Switzerland

**Keywords:** Cryospheric science, Natural hazards

## Abstract

Dry-snow slab avalanches result from crack propagation in a highly porous weak layer buried within a stratified and metastable snowpack. While our understanding of slab avalanche mechanisms improved with recent experimental and numerical advances, fundamental micro-mechanical processes remain poorly understood due to a lack of non-invasive monitoring techniques. Using a novel discrete micro-mechanical model, we reproduced crack propagation dynamics observed in field experiments, which employ the propagation saw test. The detailed microscopic analysis of weak layer stresses and bond breaking allowed us to define the crack tip location of closing crack faces, analyze its spatio-temporal characteristics and monitor the evolution of stress concentrations and the fracture process zone both in transient and steady-state regimes. Results highlight the occurrence of a steady state in crack speed and stress conditions for sufficiently long crack propagation distances (> 4 m). Crack propagation without external driving force except gravity is possible due to the local mixed-mode shear-compression stress nature at the crack tip induced by slab bending and weak layer volumetric collapse. Our result shed light into the microscopic origin of dynamic crack propagation in snow slab avalanche release that eventually will improve the evaluation of avalanche release sizes and thus hazard management and forecasting in mountainous regions.

## Introduction

Highly porous brittle materials subject to mixed mode (compressive-shear) loading exhibit localized progressive failure resulting in the nucleation of a closing crack that may propagate dynamically^1–3^. In snow, this process—also known as anticrack—is known to occur in weak snowpack layers, which have a peculiar and highly anisotropic structure related to their formation mechanism through temperature metamorphism^4^. When buried below a cohesive snow slab, the snowpack becomes metastable and a small perturbation may lead to crack propagation in the weak layer across the slope and subsequent snow slab avalanche release^5,6^. During the past decade, our understanding of fracture processes in snow has greatly improved by the development of a fracture mechanical field test known as the propagation saw test (PST)^7–9^. The PST involves isolating a snow column, initiating a crack by a sawing in a pre-identified weak layer until a critical crack length is reached, and crack propagation occurs without additional loading. The PST allows analyzing the onset and dynamics of crack propagation and deriving mechanical properties using displacement-tracking techniques^10,11^. In addition, analytical and numerical models based on fracture and/or continuum mechanics were developed to investigate crack propagation and avalanche release^3,12–17^.

These models provided new insight into key parameters and driving forces, however, the micro-mechanical processes involved during dynamical crack propagation are still essentially unknown. While their direct observation is so far not feasible, the discrete element method (DEM) has previously been successfully used to study the influence of snow microstructure on the mechanical behavior of snow^18–21^ and crack propagation in weak layers^15,22^. Therefore, DEM is an appealing method to study the effect of the complex and highly porous snow microstructure on the dynamics of crack propagation, which does not require the assumption of a complex macroscopic constitutive model. DEM allows the generation of highly porous samples crucial to model snow failure and was, for instance, used to perform 2-D simulations of a PST yielding good agreement with field experiments. However, the oversimplified shape (triangular structure) and the 2-D character of the weak layer employed by Gaume et al.^15^ prevented a detailed analysis of the internal stresses during crack propagation.

Our aim is therefore to numerically simulate an exemplary experimental PST with a 3-D DEM model to better understand the micromechanics involved during dynamical snow fracture. We first present a method to evaluate the location of the crack tip, which is particularly challenging due to the closure of crack faces during propagation. Our model reproduced the experimentally observed displacement field, accelerations and crack propagation speed well. Furthermore, the model provides detailed insight into the micro-mechanical processes and stresses within the weak layer and allows us to identify the main drivers of crack propagation.

## Methods

### Experimental data

The experimental data we use for model development were obtained with a field experiment, a propagation saw test (PST), on a flat and uniform site close to Davos, Switzerland; the experimental procedures are described in detail by Bergfeld et al.^11^. The PST is a fracture mechanical test for snow^7,23^, consisting of isolating a 30 cm wide snow column of variable length over the entire snowpack height containing a buried weak layer. While the standard guideline^24^ recommend a column length of 1.20 m, in our experiment the PST was 4.35 m long. The snowpack was characterized with a traditional manual snow profile, providing information on density, layer thickness and snow grain type.

The side wall of the PST was speckled with black ink to increase the contrast and perform digital image correlation (DIC) analysis. The experiment was filmed using a high-speed camera (Phantom, VEO710) at 14,000 frames per second and a full width resolution of 1280 pixels. The DIC analysis provided high-resolution slab, weak layer and substratum displacements^11^. Table 1 summarizes some key properties of the PST experiment. The critical crack length (${a}_{c}$) is the length of the artificial crack created by sawing through the weak layer when self-propagation starts. Mean slab density was obtained from the thickness weighted layer densities (measured manually). Snow samples including the weak layer (0.30 m × 0.30 m × 0.30 m) were transported in an insulated foam box from the test site to the cold laboratory where weak layer density was derived from micro-computed tomography^25^. The crack propagated to the very end of the column (PST fracture type: END), although a slab fracture (SF) occurring towards the end of the column was revealed by the DIC analysis.

**Table 1:** Properties of PST experiment.

Mechanical property	Macroscopic
Slope angle (degree)	0
Mean weak layer density (kg m^−3^)	138
Mean slab layer density (kg m^−3^)	154
Weak layer thickness (m)	0.02
Slab layer thickness (m)	1.1
PST fracture type	SF + END
Critical crack length ${a}_{c}$ (m)	0.32

### Discrete element method and contact model

To generate a model of the propagation saw test, we used the three-dimensional discrete element method (DEM). DEM, first introduced by Cundall and Strack^26^, is a numerical tool, consisting of a large number of discrete interacting particles, commonly employed to study large deformations in granular-like assemblies. We used the PFC3D (v5) software (http://www.itascacg.com).

The particle contact law we used is called a parallel-bond contact model (PBM) introduced by Potyondy and Cundall^27^. The PBM provides the mechanical behavior of a finite-sized piece of cement-like material connecting two particles. The PBM component acts in parallel with a classical linear contact model and establishes an elastic interaction between the particles. The PBM mechanical parameters include the contact elastic modulus, Poisson's ratio, restitution coefficient, and the friction coefficient$.$ If particles are bonded, the bond part will act in parallel to the contact part. The bonded part is described by the bond elastic modulus, the bond Poisson’s ratio and the bond strength; shear and tensile strength (“Appendix 3” illustrates the PFC parallel bond model (PBM) with the mechanical parameters for the bonded and unbonded state and the different bond behaviors e.g.; tension–compression, shear, bending and torsion). Bobillier et al.^18^ related PBM contact model parameters to macroscopic snow parameters. The PST model include macroscopic mechanical and particle parameters. The macroscopic mechanical response was obtained with numerical load-controlled shear and compression tests as presented by Bobillier et al.^18^ and summarized in Table 2.

**Table 2:** Mechanical properties of DEM model.

Mechanical property	Macroscopic	Particles
Poisson’s ratio ${\upsilon }_{u}$	−	0.3
Restitution coefficient ${e}_{u}$	−	0.1
Friction coefficient ${\mu }_{u}$	−	0.5
Mean weak layer density (kg m^−3^)	130	650
Mean slab layer density (kg m^−3^)	154	280
Slab porosity	45%	−
Weak layer porosity	80%	−
*Slab elastic modulus (MPa)	5.2	7
*Weak layer elastic modulus (MPa)	1	47.5
*Slab tensile strength (kPa)	Infinite	Infinite
*Weak layer tensile strength (kPa)	2	144
*Weak layer shear strength (kPa)	1.2	144
*Weak layer compressive strength (kPa)	4.3	−

An asterisk (*) indicates those properties that were selected by optimizing the agreement between experiment and simulation.

### System generation

The simulated three-dimensional PST consisted of three layers: a rigid basal layer, a weak layer (porous and anisotropic structure) and a slab layer (dense and uniform structure). The basal layer was composed of a single square matrix layer of particles with a radius of *r* = 2.5 mm. The weak layer was created by cohesive ballistic deposition^18,28^ resulting in a porosity of 80% for a particle radius *r* = 2.5 mm with a layer thickness of *h* = 20 mm. The slab layer was generated by cohesionless ballistic deposition^18,29^. The porosity of the slab layer was 45% for a particle radius of *r* = (10.5 ± 0.5) mm (uniform distribution) and slab thickness was *H* = 1.1 m, as in the experiment. The variation in radius was introduced to prevent close packing. The generated weak layer microstructure is unique but applying homothetic transformation would change its thickness while keeping its mechanical behavior^18^ (Figure supplement). Slab and weak layer fabric tensors were computed and there eigenvalues used to evaluate the degree of anisotropy (spheres: $\lambda_{1} \approx \lambda_{2} \approx \lambda_{3}$, discs: $\lambda_{1} \approx \lambda_{2} \gg \lambda_{3}$, and rods: $\lambda_{1} \gg \lambda_{2} \approx \lambda_{3}$). Benn^30^ defined an isotropy index (I) and an elongation index (EI) to describe the fabric shape as:

$$I = \frac{{\lambda_{3} }}{{\lambda_{1} }};{ }EI = 1 - \left( {\frac{{\lambda_{2} }}{{\lambda_{1} }}} \right).$$

Slab isotropy index and elongation were similar to values reported for layers of rounded grains ($I = 0.94;EI = 0.08$); the weak layer shows transverse isotropic symmetry similar to layers of depth hoar, surface hoar or facets ($I = 0.75;EI = 0.25$)^31^. Particle densities were adjusted to fit the observed macroscopic densities in accordance with the macroscopic sample porosities (Table 2).

**Figure 1:**
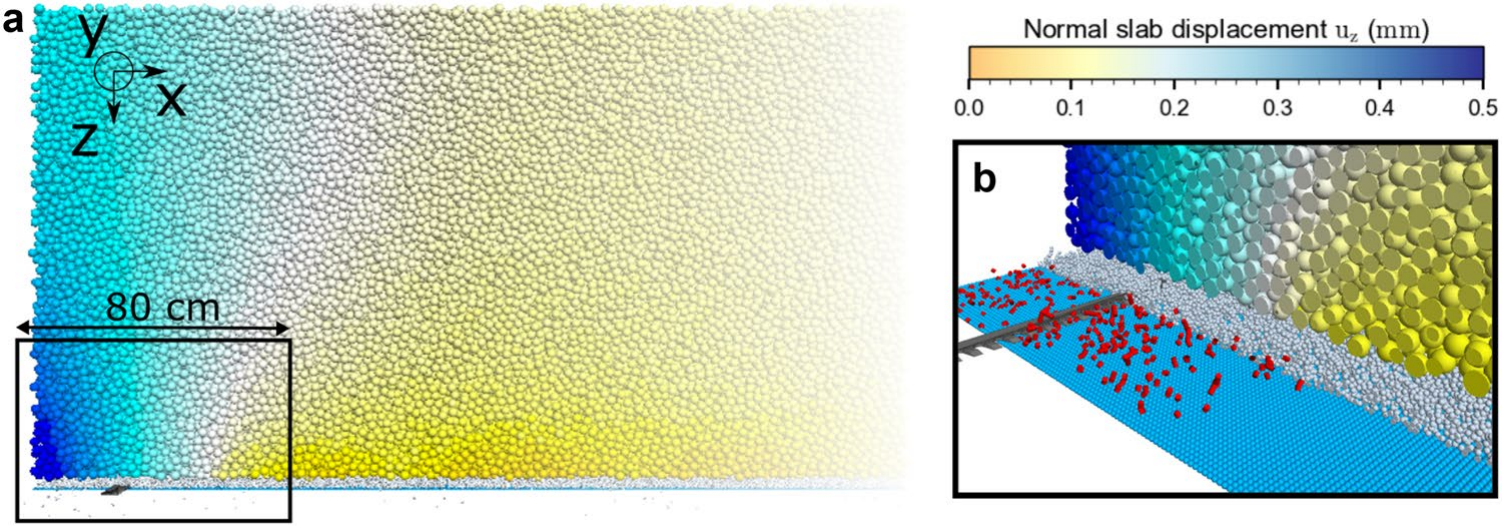
(**a**) Simulation of PST at the beginning of self-sustained crack propagation. The basal layer is represented in light blue (*r* = 2.5 mm), the weak layer (WL) in white (*r* = 2.5 mm), and the slab layer is colored by its normal displacement [colored from no displacements in yellow to 0.5 mm displacement in blue, *r* = (10.5 ± 0.5) mm]. The snow saw is represented in dark grey. (**b**) Internal WL fracture process around the fracture process zone (WL and slab were removed from the visualization). Red necks represent broken bonds in the weak layer. At the beginning of self-sustained crack propagation, many bonds are breaking ahead of the saw tip.

To generate the large DEM simulation domain needed to reproduce the experimental PST, we generated a cell under spatial periodic boundary conditions (base, weak layer and slab) large enough to avoid sample size effects (0.50 m × 0.50 m × 1.12 m)^18^. We then replicated and joined multiple cells (9 in total) to obtain the complete PST system (≈ 311,000 particles, ≈ 730,000 contacts). Finally, the spatial boundary conditions were defined as non-periodic and the system geometry was cut to fit the experimental set up (4.35 m × 0.30 m × 1.12 m). This generation technique allowed to rapidly generate a large system (~ 1 m^3^ per min; Intel Xeon CPU 2.60 GHz, 14 Cores, RAM 256 Gb). The snow saw was modeled with a PFC3D rigid wall with dimensions similar to the saw used in the field experiment (400 mm × 35 mm × 3 mm, Fig. 1; Figure supplement). Following the standard PST guideline, the saw opened a crack while cutting the weak layer at a constant speed of (1 m s^−1^). The saw speed selected was twice as high as the experimental speed to reduce the simulation time, but was still small compared to the crack propagation speed. The saw cut causes local forces in the weak layer that break bonds at the saw tip.

### Simulated PST data acquisition

From the DEM simulations, we retrieved particle displacements, forces acting on particles and the positions of broken bonds. The displacement of the slab was obtained from particle displacements and averaged over cells of 20 mm × 300 mm × 20 mm. The stresses ($\sigma_{zz} ,\tau_{zx}$) were calculated from the measured forces at the interface between the basal layer and the weak layer as:

$$\sigma_{zz} \left( x \right) = \frac{1}{{\left( {l_{y} \Delta_{x} } \right)}}\mathop \sum \limits_{n = 1}^{{n_{x} }} F_{z}^{\left( n \right)} \left( x \right)$$

$$\tau_{zx} \left( x \right) = \frac{1}{{\left( {l_{y} \Delta_{x} } \right)}}\mathop \sum \limits_{n = 1}^{{n_{x} }} F_{x}^{\left( n \right)} \left( x \right)$$

$$n_{x} :number\:of\:contacts\left[ {x;x + \Delta_{x} } \right]$$

The basal layer was discretized over the column length in order to determine the stresses (Δ_x_ = 20 mm, 8 particles).$F_{z}$ and $F_{x}$ are the contact forces acting on the basal layer in the normal and tangential direction, respectively, and $\left( {l_{y} \Delta_{x} } \right)$ is the area of the basal layer where the forces are measured ($l_{y}$ correspond to the PST width: 0.30 m).

The positions of broken bonds were recorded from the difference in the number of bonded contacts between two time steps, the missing bonded contacts are defined as broken bonds and their corresponding positions are recorded.

Crack speed was defined using $c = \Delta d/\Delta t$ where $\Delta d$ is the difference in crack tip position (for definition see below) between two time steps and $\Delta t$ the time step. To increase the precision of the data without distorting the signal tendency the finite difference method was used to approximate the derivative of the crack tip position as function of time employing Savitzky–Golay filtering. Crack speed was computed and filtered with a 9-point linear Savitzky–Golay filter^32^.

### Selection of material properties

While the slab and weak layer density were obtained during the field experiment, we needed to assume the elastic modulus and shear strength for both layers based on literature values^10,31,33^. To match the dynamics of slab displacement during crack propagation (collapse height, slab displacement speed), we simulated PSTs with different pairs of elastic moduli ($E_{{{\text{slab}}}}$, $E_{{{\text{wl}}}}$) selected within the range of the typical literature values corresponding to the layer densities we measured in the experiment ($E_{{{\text{slab}}}}$ range 4–6 MPa; $E_{{{\text{wl}}}}$ range 0.5–1 MPa). We also tuned the weak layer shear strength ($\tau_{wl}^{th}$) to match the experimental critical crack length ($a_{{\text{c}}}$) and the collapse height with respect to the admissible range given by the measured mean density of the weak layer ($\tau_{wl}^{th}$ range 1–2 kPa).

### Onset of crack propagation

The critical length of the saw cut, i.e. the onset of self-sustained crack propagation, was measured as the length of the open crack induced by sawing. The critical length measured in a field experiment is subject to inherent uncertainties induced by the manual sawing (on the order of ± 5 cm). In the simulation, we arbitrarily defined the critical cut length as the saw position when at least one bond was broken 0.20 m in front of the saw tip. Using a realistic value for the weak layer shear strength of 1.2 kPa^34^ we obtained a critical cut length in the numerical simulation of *a*_c_ = 0.28 m, which is shorter than in the experiment (*a*_c_ = 0.32 m).

### Crack tip definition

**Figure 2:**
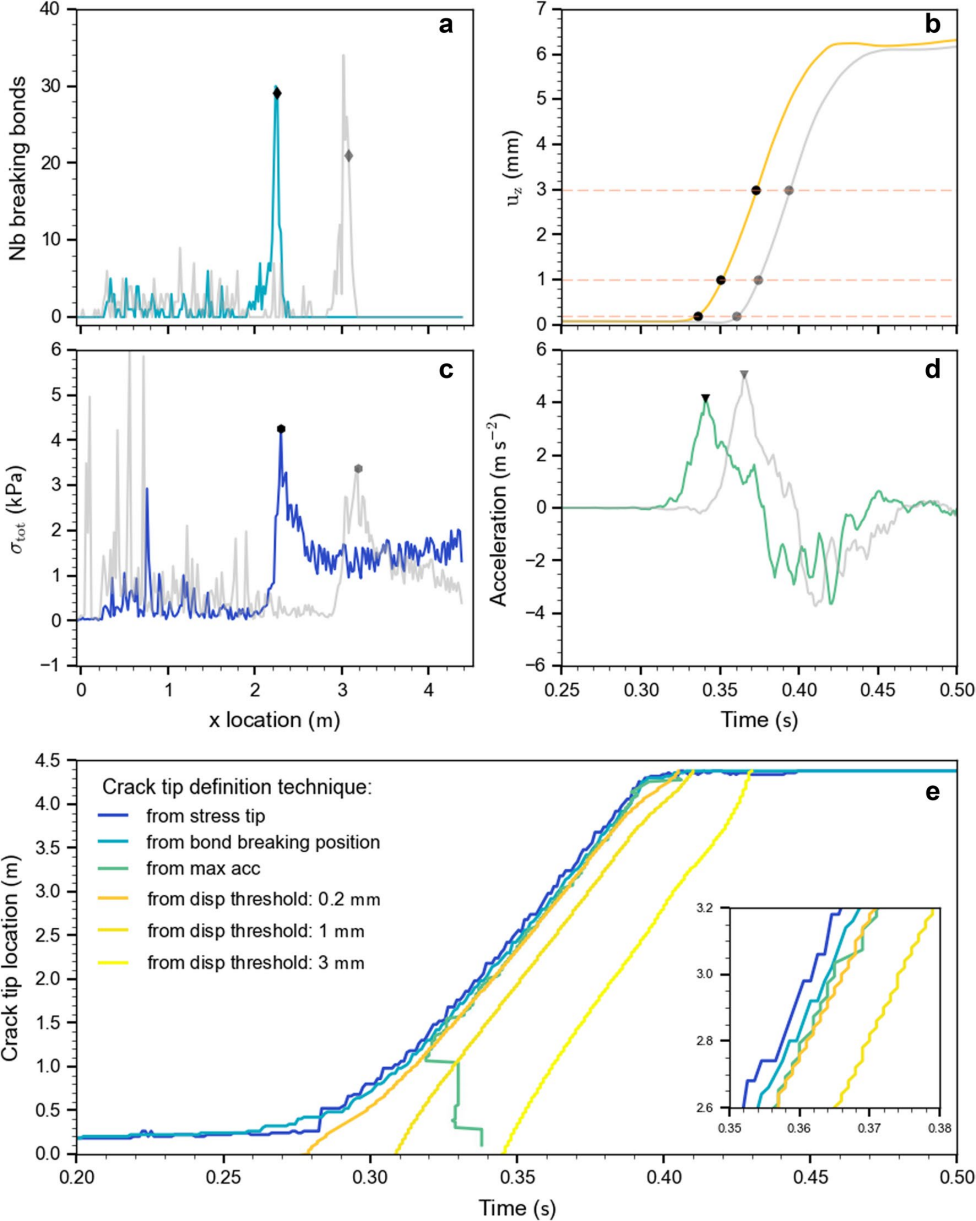
(**a**) Number of bonds breaking at a given time step along the length of the column; distributions for two different time steps shown (0.28 s and 0.3 s). (**b**) Temporal evolution of the normal slab displacement for two locations (x = 2 m and 3 m). The orange dashed horizontal lines correspond to three displacement thresholds (0.2, 1 and 3 mm). (**c**) Total stress along the length of the column at two different time steps (0.34 s and 0.36 s). (**d**) Temporal evolution of the slab normal acceleration for two x locations (at 2 m and 3 m). Markers show the position or time obtained to define the crack tip (in **a**, **b**, **c**, **d**). (**e**) Temporal evolution of the crack tip position for different metrics of crack tip definition. (**f**) Inset shows zoom into the temporal evolution of the crack tip position.

To accurately determine crack speed, it is crucial to define the position of the crack tip. This is not straightforward since we do not have an opening crack, but a closing (anti-)crack. Hence, we suggest four metrics for determining the position of the crack tip: (a) bond-breaking position, (b) normal displacement thresholds, (c) maximum stress, and (d) maximum normal slab acceleration (Fig. 2). Definition (a) is based on the number of broken bonds at a given time step. Starting from the end of the column, at each time step the crack tip is defined as the position where the number of broken bonds corresponds to 70% of the maximum number of broken bonds (Fig. 2a: diamond dots). Definition (b) is based on a normal displacement threshold as suggested by van Herwijnen and Jamieson^9^. For every subset, the time is recorded when the displacement exceeds the threshold value; to compute the speed, $\Delta_{d}$ is equal to the discretization length ($\Delta_{x} = 20 {\text{mm}}$) and $\Delta_{t}$ is the recorded time difference (Fig. 2b). Figure 2e shows the temporal evolution of the crack tip position defined with three different threshold values of normal displacement (three shades of yellow). Increasing the threshold value shifts the crack tip position in time. In definition (c), the crack tip is the position of maximum total stress ($\sigma_{tot} = \sqrt {\sigma_{zz}^{2} + \tau_{xz}^{2} } ;$ Fig. 2c). Finally, in definition (d), the crack tip is the position of maximum normal acceleration as a function of time (Fig. 2d). Figure 2e, f shows good agreement between the four methods of crack tip definition; the crack speed between 1 m and the end of the column shows the same behavior regardless of the type of metric used. The crack tip location based on the maximum stress definition is ahead of the locations obtained with the other methods (Fig. 2e, f) in line with the assumption that stresses induce fracture. The crack tip definition based on the bond breaking position provides the maximum of information during the cutting phase. While the metric based on acceleration (d) was used to define the crack tip position, due to noise in particle acceleration shortly after the onset of crack propagation, it could not be used to reliably compute crack speed.

### Time step

The length of the time step was determined as function of the particle properties according to

$$\Delta t \approx {\text{f r}}\sqrt {\frac{\rho }{E }}$$

where $\rho$ and $r$ are the smallest particle density and radius, respectively, $E$ is the largest bond or particle elastic modulus, and *f* is a safety factor (0.8). PFC3D software dynamically calculated the time step in this manner to ensure the stability of the DEM.

## Results

### Dynamics of crack propagation and crack propagation speed

**Figure 3:**
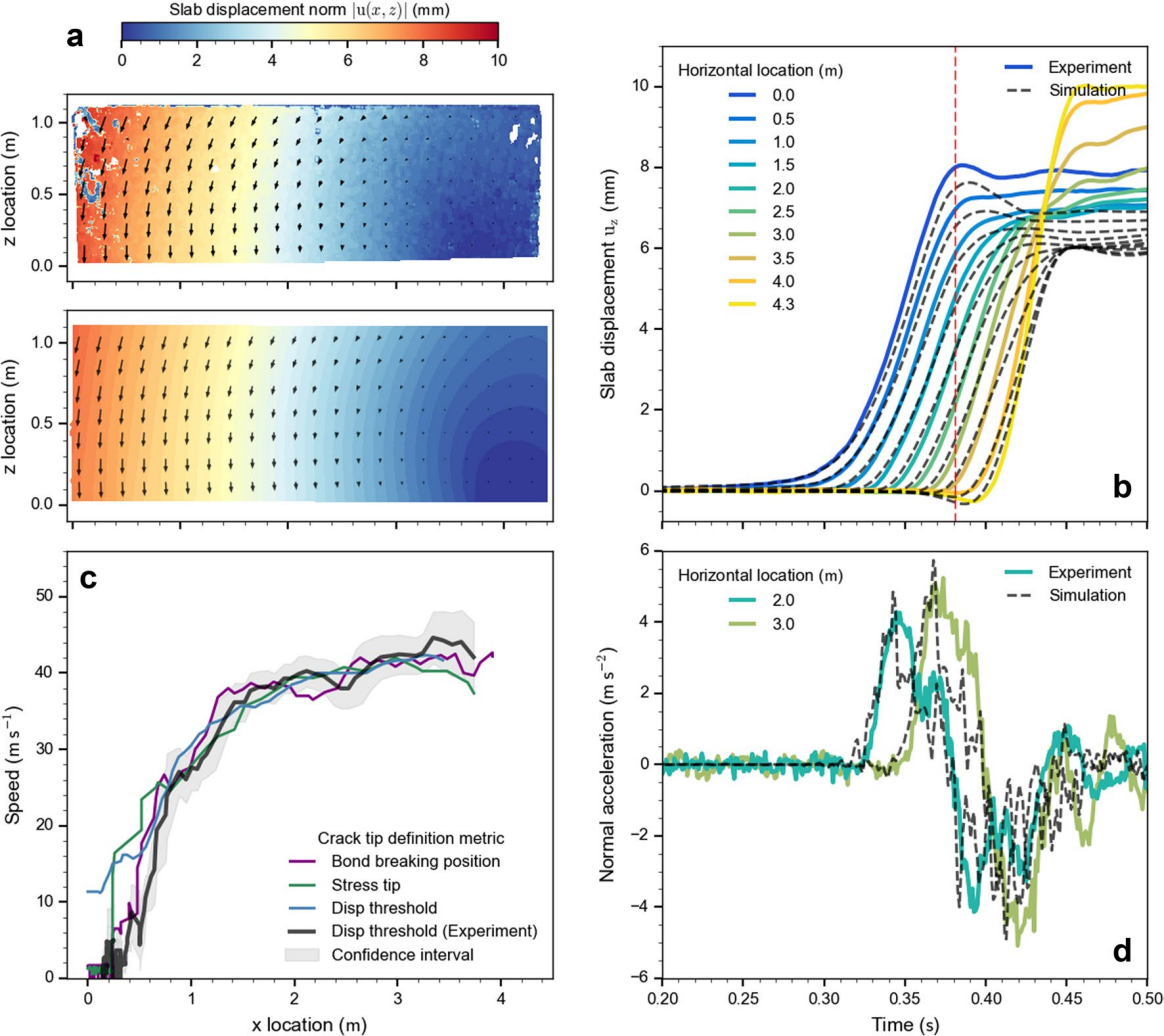
Comparison of experimental and simulated PST results. (**a**) Experimental (top) and simulated (bottom) displacement field norm (magnitude) when the crack tip reached 3.7 m ($\left|u\left(x,z\right)\right|=\sqrt{{{u}_{x}}^{2}+{{u}_{z}}^{2}}$). The displacement field is colored from no-displacement in blue to 10 mm displacement in red. (**b**) Temporal evolution of the normal displacement averaged over the height. The colors represent the horizontal location and the black dashed line the corresponding location for the simulated PST. The red dashed line corresponds to the time when the crack tip reached 3.7 m, i.e. corresponds to the displacement field shown in (**a**). (**c**) Crack speed evolution along the PST beam. The grey line shows the experimental crack speed with its confidence interval (grey envelope) and the blue, violet and green lines the simulated crack speed. Crack speeds are computed from different crack tip definitions: grey and blue line based on displacement threshold of 0.2 mm, green line based on maximum stress and violet line based on the position of breaking bonds. (**d**) Temporal evolution of the normal slab acceleration for two locations [at 2 m and 3 m; line colors as in (**b**)].

The dynamics of key measures observed in the PST field experiment were very well reproduced by the DEM simulation. The displacement field exhibited the same behavior in the experimental and the simulated PST (Fig. 3a; Supplementary Movie 1). At the time step when the crack tip reached 3.7 m, the column end was still in its initial state while the displacement was maximal at the front part of the column, which allows comparing the ‘bending’ behavior over the entire beam. The temporal evolution of slab subsets averaged over the height located every 0.5 m showed also good agreement between experimental and simulated PST (Fig. 3b). For all locations shown along the beam, the small slab displacements (< 1 mm), which correspond to weak layer failure, were very closely reproduced. Normal slab accelerations of subsets averaged over the height located at 2 and 3 m agreed well between experimental and simulated PST (Fig. 3d). The large slab displacement in Fig. 3b corresponds to the weak layer; we note that from 3.5 m the collapse height in the experiment was larger than in the simulation, in part due to a slab fracture observed in the experiment (“Appendix 1”).

Crack speed along the PST beam was computed from experimental and simulation data based on the different metrics to define the crack tip location (Fig. 3c). The crack speed evolution revealed four phases. First, during the cutting phase, the crack evolved at the same speed as the saw (1 m s^−1^ in the simulation). Second, a transitional regime originated when the crack started to self-propagate and speed quickly increased over a distance of approximately 1.6 m until it reached a plateau. Third, up to the beam end, the crack speed was relatively constant around 40 m s^−1^ in an apparent steady-state regime. Finally, in the last phase, the experimental crack speed slightly increased, which does not show in the simulation.

### Mechanical analysis of crack propagation

**Figure 4:**
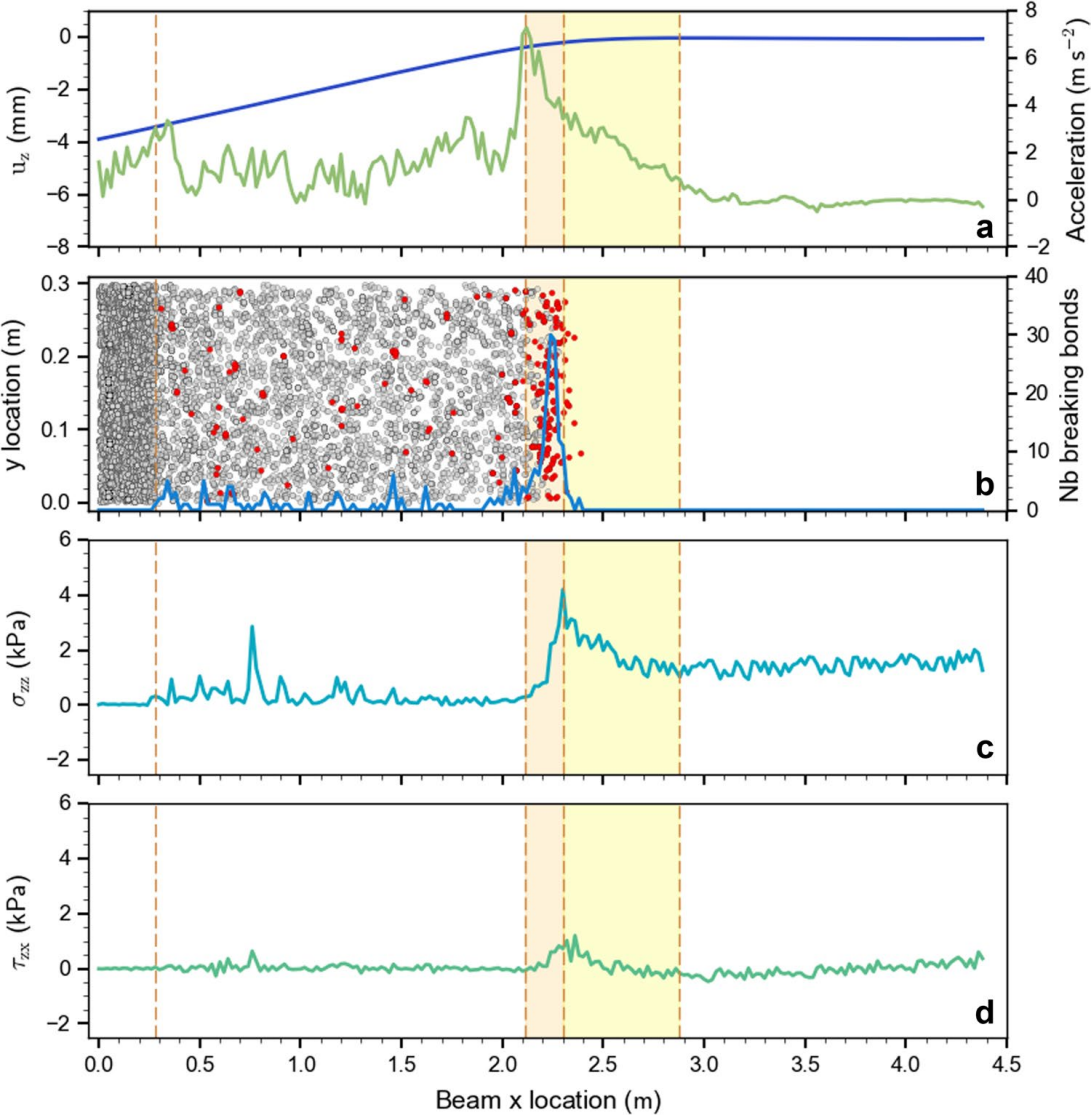
Mechanical parameters during crack propagation: snapshot at 0.344 s (after the start of the simulation). (**a**) Average normal slab displacement in blue and average normal slab acceleration in green. (**b**) Top view of the weak layer showing the bond states: in grey broken bonds, in red bonds that are breaking at current time step. The blue line shows the breaking bond distribution along the length of the beam. (**c**) Normal stress ${\sigma }_{zz}$ and (**d**) shear stress ${\tau }_{zx}$ along the length of the beam. The light red box highlights the fracture process zone (FPZ). The light yellow box highlights the part of the beam where the stresses are redistributed. The orange vertical dashed lines indicate the start and end of the different sections (1, 2, 3, 4, and 5).

During the simulation, we tracked micro-mechanical quantities: the position of bond-breaking events, normal displacement and acceleration as well as shear and normal stresses at the top of the basal layer. The temporal evolution of these quantities revealed six distinct sections during the fracture process (Fig. 4, labeled at the top from 1 to 5). In the following, we describe five of the six sections for the situation shown in Fig. 4, i.e. when the crack tip is at about at 2.3 m, corresponding to a simulation time of 0.344 s.

1. In the saw cut area, from 0 to 0.28 m, there are numerous broken bonds (grey dots in Fig. 4b) and the stresses are low.
2. From 0.28 to 2.12 m, there are many broken bonds and a few bonds breaking at the current time step (grey dots and red dots in Fig. 4b, respectively); stresses remain low as the weak layer already fractured and the slab is subsiding, but not yet resting on the crushed weak layer. A few stress peaks indicate that some highly loaded contacts exist, but those disappear in the next time step confirming that the slab is still subsiding and the weak layer structure is collapsing (see Supplementary Movie 2).
3. The section from 2.12 to 2.3 m, represents the fracture process zone (FPZ; light red box in Fig. 4) where most of the bonds are breaking in a narrow zone (Fig. 4b) and stresses reflect a mixed-mode loading state (shear-compression) where the compressive (normal) stress is the dominant driver. The end of the FPZ corresponds to the maximum normal stress. In the FPZ, the concave stress shape indicates that the weak layer softens ^35^.
4. From 2.3 to 2.87 m, the weak layer is mostly undamaged (light yellow box in Fig. 4) and stresses decrease until reaching far-field values induced by gravity. In this zone, stresses are governed by elastic redistribution over a characteristic length scale Λ, depending on slab and weak layer elastic properties. Gaume et al.^22^ suggested Λ to be given as follows: Λ = (*E*_*slab*_*'* *H*_*slab*_ *H*_*wl*_/*G*_wl_)^1/2^ where *E*_slab_*'* = *E*_slab_/(1 – *ν*^2^) is the plane stress elastic modulus of the slab and *G*_wl_ the weak layer shear modulus (*G*_wl_ = 0.21 MPa). In our case, the theoretical value is Λ = 0.71 m, in reasonable agreement with the distance from the maximum stress to the location where the stresses reach the initial state value (Fig. 4c, d; light yellow box; length: 0.57 m). Hence, the elastic redistribution length is about twice the length of the FPZ.
5. From 2.87 m to the end of the beam no bonds are breaking, the normal stress is equal to the gravitational stress, and the shear stress is zero except towards the end of the beam where it becomes slightly positive due to the free boundary condition (Fig. 4).

A sixth section appears later in the fracture process when slab comes to rest on the crushed weak layer. Stresses in this contact area fluctuate and the acceleration becomes negative (deceleration) before reaching zero when slab displacement stops (Supplementary Movie 2 and Fig. 3d). When the crack tip approaches the PST end (3.5 m), the momentum at the free edge of the beam induces negative normal stress (tension) and causes bond breaking in tension beyond the crack tip. Normal slab displacement and acceleration (Fig. 4a) exhibit specific trends for each section: large displacement in the sawing and weak layer structural collapse sections (1, 2) with a nearly constant acceleration of 2 m s^−2^, small displacements in the fracture process zone where the acceleration reaches its maximum, no displacement in the elastic redistribution section where the acceleration decreases to zero, and no displacements and accelerations in the intact section of the PST (Fig. 4a).

**Figure 5:**
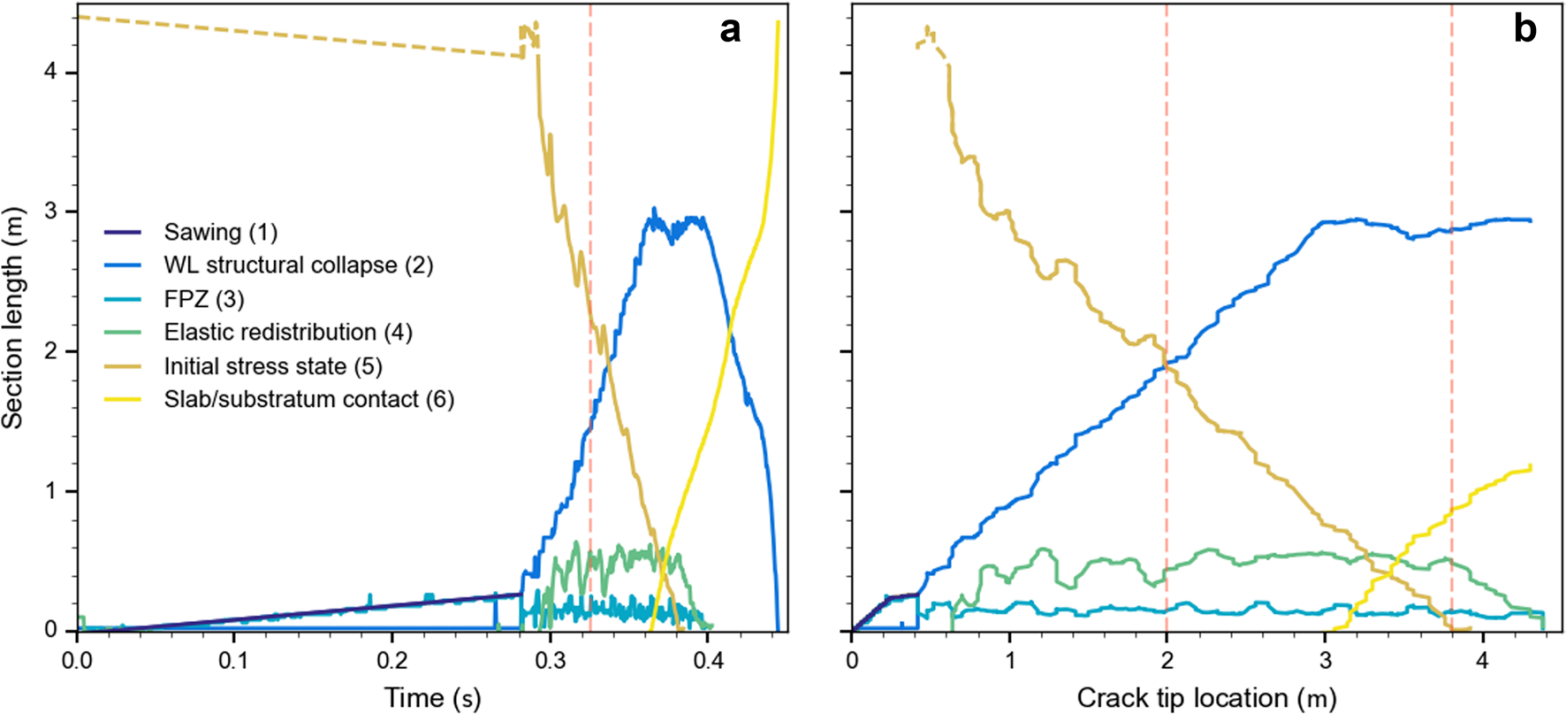
Temporal evolution of the length of six different fracture sections during a PST experiment as (**a**) a function of time and (**b**) a function of crack tip location: (1) the sawing section, (2) the section where the weak layer structure collapses, (3) the fracture process zone, (4) the elastic redistribution section, (5) the section where the system is in its initial stress state, (6) the section where the slab and the substratum are in contact. The light violet line corresponds to the sum of section lengths (2), (3) and (4). The orange vertical dashed line shows the time when the crack speed reached a plateau with constant value of about 40 m s^−1^ (beyond 1.6 m in Fig. 3c).

To investigate crack propagation from a micro-mechanical perspective, we investigated the evolution of the six sections described above in more detail along the beam (Fig. 5; see also Supplementary Movie 2). The sawing length (1) grew at the speed of the saw (1 m s^−1^) and stopped at the critical cut length *a*_c_. The section where the weak layer structure collapses (2) started when the critical cut length was reached and grew linearly until the slab came to rest on the crushed weak layer (at 0.362 s). Subsequently the length (2) fluctuated around (2.70 ± 0.05) m until the weak layer was completely fractured (at 0.394 s) and decreased rapidly. The fracture process zone (3) increased linearly during the sawing phase and then fluctuated around (0.14 ± 0.04) m during crack propagation until the crack tip reached the beam end. The length of the elastic redistribution section (4) was defined from the maximum stress to the beginning of the slab motion (arbitrarily defined as the position where the normal slab acceleration exceeds 0.85 m s^−2^). The elastic redistribution length (4) increased until the crack speed reached a constant value. It fluctuated around (0.52 ± 0.05) m (Fig. 5, orange dashed lines) before decreasing again towards the end of the experiment. The length of the section where the beam was in its initial state (5) decreased with the same speed as the saw up to the critical cut length, and then decreased more rapidly with approximately a constant speed until 0.38 s. The contact length (6) corresponded to the length where the slab rests on the crushed weak layer. It increased almost linearly once the crack tip was at 2.98 m (0.361 s).

When the crack speed was in a steady state (orange dashed lines in Fig. 5), we observed a constant length of the fracture process zone (3) and the elastic redistribution section (4). Figure 5b shows these section lengths as function of crack tip position, the lengths (2), (3) and (4) clearly exhibit a plateau at the same time confirming a steady state stress regime during crack propagation.

## Discussion

We developed a 3-D discrete element model to investigate the micro-mechanical processes at play during crack propagation in snow fracture experiments. Microscopic model properties were calibrated based on macroscopic snowpack quantities using the method developed by Bobillier et al.^18^. The field data of a PST fracture experiment was recorded during winter 2019 and analyzed with image correlation techniques^11^. The experiment provides bulk snow properties and the displacement field during crack propagation and allows studying mixed-mode failure of a porous weak snow layer. Our DEM model of the PST accurately reproduced the observed dynamics of crack propagation including the structural collapse of the weak layer. Moreover, our PST model provides insight into the micro-mechanics of the failure processes before and during self-sustained crack propagation.

Geometrical and mechanical properties retrieved from the PST experiment were used as input of our numerical model. However, as weak layer parameters cannot be measured in detail, the mechanical parameters of the layers ($E,{\tau }^{\mathrm{th}}$) were tuned to reproduce the experimental behavior. The comparison of the displacement fields (normal and tangential) shows our model can reproduce the experimentally observed displacements and slab acceleration (Fig. 3; Appendix 2). However, some discrepancies can be noted, in particular at both edges of the PST experiment. Due to the very thick slab (*H* = 1.1 m), the edges of the PST were subject to high deformation, very likely leading to plastic deformation. Furthermore, a slab fracture occurred at the far end of the PST (Appendix 1). As in our model, the slab is purely elastic, there cannot be any non-recoverable deformation. The slab fracture induced a large vertical movement causing a divergence between the displacement fields of experiment and simulation. Whereas the slab fracture observed in the experiment influenced the final slab displacement, it did not affect crack propagation behavior so that our analysis of the failure processes in the weak layer is not affected either. A simulation including slab brittle fracture is provided in Appendix 1 and shows that the model can also reproduce the complex interplay between weak layer and slab fracture.

For closing crack faces under compression (anticrack), the crack tip is not clearly defined and no formal and well-accepted definition exists. Hence, we introduced four different metrics to determine the position of the crack tip, based on slab displacement threshold, maximum normal slab acceleration, stress maxima and the distribution of breaking bonds (Fig. 2). We then computed the crack speed based on these crack tip definitions, resulting in very similar values (Fig. 3c). Over the length of the experiment, crack speeds exhibit two phases: an initial transitional regime with rapidly increasing crack speed, a steady-state regime between 1.6 m and 4 m. These phases were observed in both the numerical as well as the experimental PST.

The length of the column in our PST experiment is at least twice as long as previously reported column lengths and should be sufficient for reaching a steady state propagation^36^. We showed that after 1.6 m the crack speed starts to vary around a constant value. Even if the crack speed evolution suggests a steady state propagation, the slab fracture observed in the experiment likely influenced crack propagation, casting some doubt on the validity of this observation. Nevertheless, the experimental length combined with the uniform collapse height support the interpretation that crack propagation reached a steady state. The mean value observed during the steady state speed phase (approx. 40 m s^−1^) is of the same order of magnitude as previously reported propagation speeds^9,37,38^. However, a direct quantitative comparison is hindered by the fact that previously reported propagation speeds were primarily computed for relatively short PSTs (< 2 m) and speeds were averaged over the length of the beam.

Heierli^39^ proposed an analytical model and suggested that the crack, in the weak layer, occurs in the form of a localized disturbance zone propagating as a collapse wave with constant speed $c_{h} ~ = ~\sqrt[4]{{ - \frac{g}{{2h}}\frac{D}{{\rho H}}}}$. Here $D$ is the flexural rigidity $D = \frac{{E_{slab} H^{3} }}{{12\left( {1 - {\upnu }^{2} } \right)}}$, *g* the gravitational acceleration, *h* the collapse height, $\rho$ the mean slab density, *H* the slab thickness and ${\upnu }$ the Poisson’s ratio. In the presented PST model, this bending wave would propagate with $c_{h} = 40.9 \,{\text{m }}\,{\text{s}}^{ - 1}$. For comparison, the slab longitudinal wave: $c_{l} = \sqrt {E/\rho } = 183{ }\;{\text{m}}\;{\text{s}}^{ - 1}$, and the slab shear wave speed: $c_{s} = \sqrt {G/\rho } = 122 {\text{m s}}^{ - 1}$. The formulation suggested by Heierli^39^ agrees well with the measured steady state speed, longitudinal and shear wave speeds are much higher; nonetheless, we cannot easily interpret the measured crack speed. A future sensitivity study may provide more insight.

The DEM model of the PST experiment allows insight into the micro-mechanical behavior of weak layer failure. We suggest six sections to describe the crack dynamics during a PST experiment; (1) sawing, (2) weak layer collapse, (3) fracture process zone, (4) elastic redistribution, (5) undisturbed (initial) stress state, and slab-substratum contact (6). We looked into three of these sections in more detail: (2) The structural weak layer collapse (crushing) where the stresses remain low and only a few bonds are breaking. (3) The fracture process zone where the material softens and most of the bonds are breaking and where the stress is maximal. (4) The elastic redistribution zone where the stresses are converging to the initial undistributed stress state and no bonds are breaking. During steady state propagation, we observed that these three sections travel along the beam keeping their behavior, which is defined by the geometrical and mechanical properties (Fig. 5; Supplementary Movie 2). Frame by frame, the stresses were analyzed and the results indicated a mixed-mode bond failure with a main normal stress component (Supplementary Movie 2). We also noted (not shown) that the PST width does not influence the crack tip morphology. Before reaching a steady state speed regime, we defined a transitional regime where we observed a decrease in the normal stress and an increase of the shear stress component.

Gaume et al.^15^ introduced a 2-D DEM model of a PST experiment to study crack propagation. Their model consisted of a single particle base layer, a triangular shape for the weak layer structure and a square particle matrix for the slab layer. Their simple model was used to study the influence of mechanical parameters on crack propagation. However, the simplistic layer representation precluded a detailed micro-mechanical analysis. The model presented here overcomes this limitation and allows insight into the micro-mechanics, yet at the expense of high computational cost. Still, to keep the computational cost reasonable (~hours to day), the particle radius chosen does not represent single snow grains, but represents a mesoscale model of snow^18^. To permit this analysis, we discretized the column length and the mesh size was driven by the particle size, which remains the main model limitation. Observing the final slab state suggested the existence of plastic deformation and slab fracture. By modifying slab tensile strength, we were able to reproduce the observed slab fracture (Appendix 1). However, the contact law we use does not allow for non-recoverable slab deformation.

The experimental slab displacement exhibits high damping behavior when the slab comes into contact with the broken weak layer (no slab bouncing). Our approach was to use a damping coefficient to dissipate slab kinetic energy avoiding spurious oscillations, therefore, reproduce slab displacement, velocity and acceleration (Figure supplement). In reality, this numerical damping may encompass different types of sources of energy dissipation that are not accounted yet, such as slab plastic hardening, mechanisms related to weak layer bond failure (quasi-brittle softening behavior), or rate-dependent effects such as fast sintering. The accumulated strain energy in weak layer bonds is released upon bond failure (perfectly brittle behavior) and leads to crack propagation. Introducing a quasi-brittle softening contact model for the weak layer and allowing slab plastic deformation are possible improvements to the physics of the model.

In this study, we only reproduced one single PST experiment performed in flat terrain. The availability of the experimental data obtained with the correlation technique as well as the computational time to tune model parameters were the key limiting factors preventing extensive model validation, which was not our primary goal. Rather we focused on bond breaking evolution and stresses to provide new insights into the propagation dynamics. These novel conceptual findings seem robust and well-founded in the model results. In the future, we will explore model sensitivity on propagation behavior and exploit the micro-mechanical insights in view of snow slab avalanche release modeling. We will focus on the effect of mechanical parameters: on the transient acceleration regime and steady-state regime, and on the micro-mechanics such as the stress concentration and bond breaking morphology. The influence of slope angle will also be studied to gain more insight into the avalanche formation mechanism.

## Conclusions

Understanding crack propagation behavior in the highly porous and anisotropic material snow is crucial to appropriately model snow slab avalanche release. In this study, we reproduced a fracture mechanical field experiment with a 3-D DEM simulation. The model results highlight the occurrence of a steady state in crack speed and stress conditions for sufficiently long PSTs (> 4 m). In our case of closing-mode (anticrack) fracture, the identification of the crack tip is not straightforward. Hence, we proposed four different methods to define the crack tip that can be applied for experimental as well as simulated data. While the different methods yield different crack tip locations, they provide very similar values of crack speed. This finding suggests that previously used displacement threshold methods on experimental data to derive crack speed are appropriate.

As our DEM approach allows insight into bond-breaking events and stresses, we suggest six sections to describe the crack dynamics during a PST experiment. In particular, three distinct sections travel along the beam while keeping their behavior during the steady state stress regime: weak layer structural collapse, fracture process zone, and elastic redistribution. The detailed micro-mechanical analysis of stresses for weak layer failure suggests that the main drivers of crack propagation is the mixed mode stress concentration at the crack tip (compression and shear).

In future, we will perform a parameter study to describe the drivers of crack speed at the slope scale leading to avalanche release. The effects of slope angle and mechanical parameters on crack propagation will be studied to eventually improve the prediction of avalanche size.

### Supplementary Information

Below is the link to the electronic supplementary material.

Supplementary Video 1.

Supplementary Video 2.

Supplementary Information 1.

## Figures and Tables

**Figure 6:**
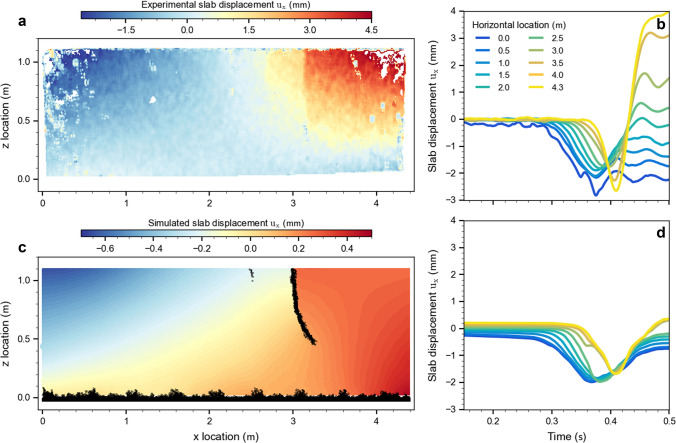
(**a**) Experimental and (**c**) simulated slab horizontal displacement field at the end of the experiment. Black dots in (**c**) indicate the positions of broken bonds and show the slab fracture. (**b**) Experimental and (**d**) simulated temporal evolution of subsets of tangential displacements located at the top of the beam; colors indicate horizontal location along the beam.

**Figure 7:**
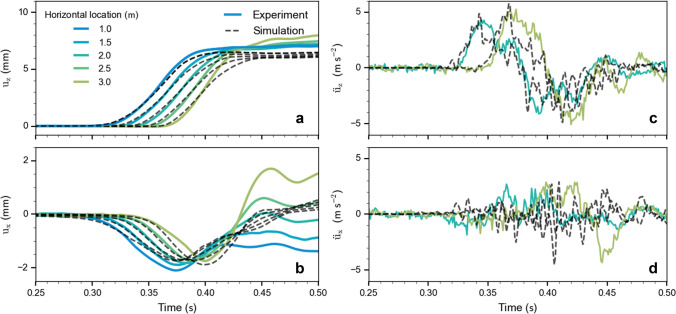
Comparison between observed and simulated displacements during PST experiment. (**a**) Temporal evolution of the normal displacement averaged over the height of the beam. (**b**) Temporal evolution of subsets of tangential displacements located at the top of the beam. (**c**) Temporal evolution of the normal acceleration. (**d**) Temporal evolution of the tangential acceleration. The colors represent the horizontal location and the black dashed lines the corresponding location for the simulated PST.

**Figure 8:**
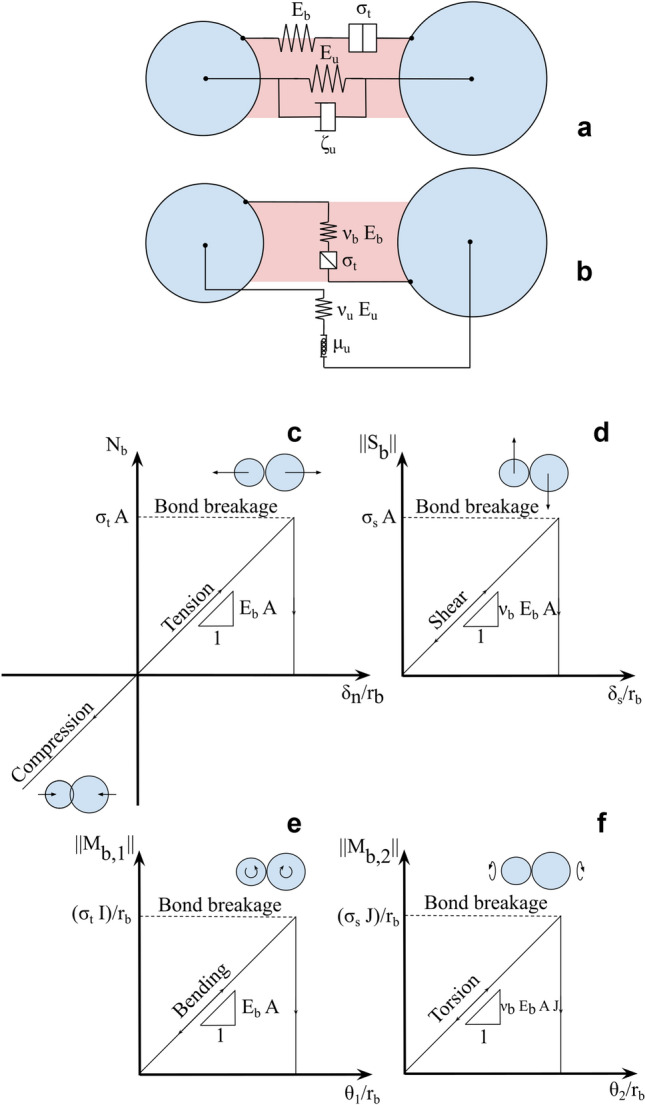
PFC3D parallel bond model (PBM) representation and its bonded behavior. (**a**) Normal mechanical parameter bonded and unbonded, where $E_{b}$ represents the bond elastic modulus, $\sigma_{t}$ the tensile strength, $E_{u}$ the contact elastic modulus and $\zeta_{u}$ the contact damping ratio. (**b**) Shear mechanical parameter bond and unbonded, where $E_{b}$ represents the bond elastic modulus, $\sigma_{s}$ the shear strength, $E_{u}$ the contact elastic modulus, $\nu_{b}$ the bond Poisson’s ratio, $\nu_{u}$ the contact Poisson’s ratio and $\mu_{u}$ the friction coefficient. To reduce the number of variables we assume $E_{u} = E_{b} \triangleq E_{particle}$, $\sigma_{s} = \sigma_{t} \triangleq \sigma_{bond}^{th}$ and $\nu_{u} = \nu_{b}$. (**c**) Bond normal force $N_{b}$ as a function of the normal interpenetration $\delta_{n}$ scaled by the bond radius $r_{b}$. (**d**) Bond shear force $\left\| {S_{b} } \right\|$ as a function of tangential interpenetration $\delta_{s}$ scaled by the bond radius $r_{b}$. (**e**) Bond-bending moment $\left\| {M_{b,1} } \right\|$ as a function of bending rotation $\theta_{1}$ scaled by the bond radius $r_{b}$. (**f**) Torsion moment $\left\| {M_{b,2} } \right\|$ as a function of twist rotation $\theta_{2}$ scaled by the bond radius $r_{b}$.

## Appendix 1: Slab fracture

The horizontal displacement field reveals the occurrence of slab fractures despite the crack travelling to the far end of the column. Figure 6a shows the normalized tangential displacement field in the final state of the experimental PST. From the 0 to 2.6 m, at the top, we observed a color continuity; at 2.6 m, the color slightly jumps from blue to yellow, which suggests a slab fracture stopped approximately 0.25 m below the top. At 3.15 m the color jumps from light orange to red; this color jump clearly indicates a slab fracture and rigid body behavior isolated from the rest of the beam. The location of the shallow, blurry slab fracture (2.6 m) corresponds to the location where the simulated and experimental normal displacement behavior start to diverge (Fig. 3b). The location of the second more distinct slab fracture (3.15 m) corresponds to the location where the simulated and experimentally observed collapse height start to diverge (Fig. 3b). In the experiment, the collapse height is larger possibly due to isolated rigid body behavior. We observed that the left and the right part of the beam kept its tangential displacement at the end of the experiment, which suggests plasticity (Fig. 6b).

For the simulation shown in Fig. 7c, the normalized tangential displacement field in the final state of the simulated experiment, we relaxed the assumption of infinite slab strength and introduced a vertical linear tensile strength gradient within the simulated slab to reproduce the slab fracture behavior (top: ${\sigma }_{slab}^{th}=$ 3 kPa, bottom: ${\sigma }_{slab}^{th}=$ 3.4 kPa). The positions of the broken bonds indicate that the observed positions of the slab fractures were qualitatively well-reproduced (black dots in Fig. 6c). Figures 6b and 7d show the temporal evolution of subsets of tangential displacements located at the top of the beam. From 0 to 2.5 m, i.e. up to 0.36 s, the experimental and simulated data show similar behavior. After 0.36 s, the simulated tangential displacement tends to return to its initial state, while in the experiment the deformation is not recovered. Figure 6d shows a clear change in behavior when the slab fractures occurred, from 3 m to the end of the column the subsets show a different behavior than at the beginning of the column. This behavior is well reproduced by the simulation until 0.38 s when the simulated displacements return to its initial state, while in the experiment the displacements are not recovered. However, unlike to the experiment, in the simulation the beam tends to recover its deformation. While adding slab strength allowed reproducing the slab fractures, the contact model does not account for plasticity. Hence, in the simulation, we cannot reproduce the final state of deformation observed in the experiment.

## Appendix 2: Comparison of slab behavior

Similar to Figs. 3b, d and 7a show the temporal evolution of the normal displacement of the slab and Fig. 7c the temporal evolution of the slab normal acceleration and (d) the slab tangential acceleration. In addition, Fig. 7b shows the temporal evolution of the tangential (horizontal) displacement of the slab. Slab behavior observed in the experiment is well reproduced by the simulation (dashed lines); the main differences appear after the so-called touchdown when the simulated tangential displacements return to its initial state, whereas the displacements in the experiment exhibit non-recoverable deformation (plasticity). This phenomenon is accentuated at the free edges of the beam and by slab fractures (Appendix 1).

## Appendix 3: Parallel bond contact model

See Fig. 8.
