# Supplementary material for: Micro-mechanical insights into the dynamics of crack propagation in snow fracture experiments
Source: Sci Rep. 2021 Jun 3;11:11711. doi: 10.1038/s41598-021-90910-3 (PMC8175457; doi:10.1038/s41598-021-90910-3)
Supplement: Supplementary file 3 — Supplementary Information 1. [file 41598_2021_90910_MOESM3_ESM.docx]

# Figure supplements


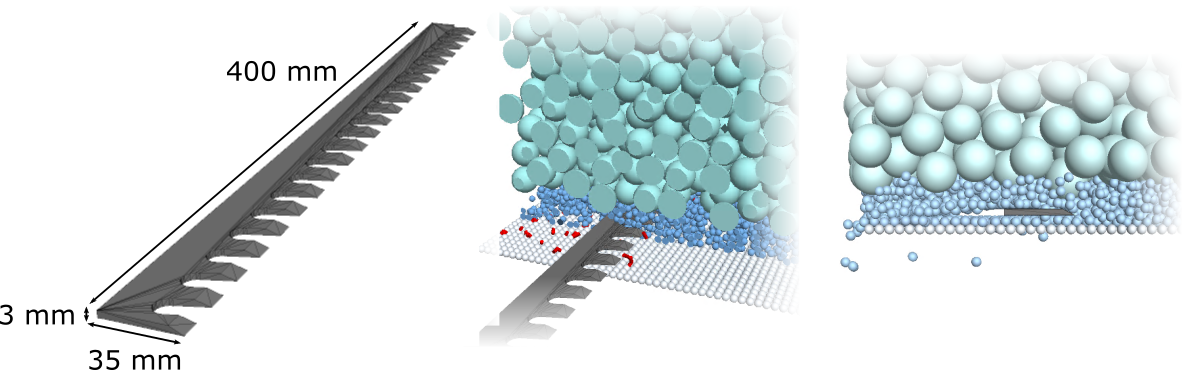


**a**

**c**

**b**

Figure 9: Simulation screenshots during weak layer cutting. (a) Modeled saw; (b) modeled snowpack and the saw (WL and slab were partially removed from the visualization). Red necks represent broken bonds in the weak layer; (c) saw cutting through the weak layer and crack formation.


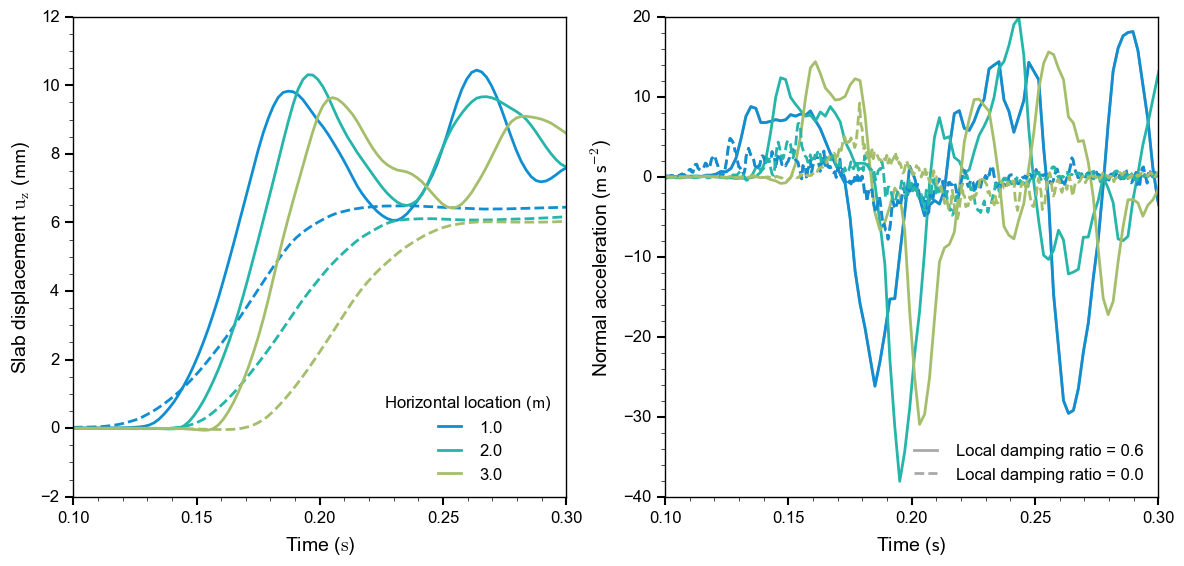


**b**

**a**

Figure 10: Local damping ratio effect on (a) slab normal displacements and (b) accelerations.

**
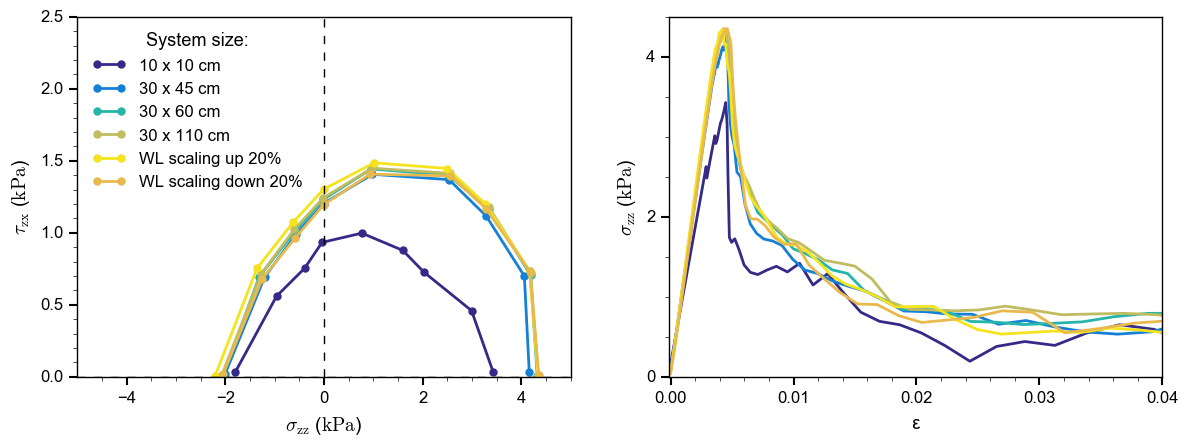
**

**b**

**a**

Figure 11: Weak layer behavior subject to load-controlled test. (a) Failure envelope for different system size and different applied homothetic transformation. (b) Normal stress as functions of the normal strain.
